# Corneal Biomechanical Changes in Third Trimester of Pregnancy

**DOI:** 10.3390/medicina57060600

**Published:** 2021-06-10

**Authors:** Inna Adriana Bujor, Raluca Claudia Iancu, Sînziana Luminiţa Istrate, Emil Ungureanu, George Iancu

**Affiliations:** 1 Department of Ophthalmology, Faculty of Medicine, “Carol Davila” University of Medicine and Pharmacy, District 5, 020021 Bucharest, Romania; moiainna@yahoo.com (I.A.B.); sanzinici@yahoo.com (S.L.I.); mdemil@gmail.com (E.U.); 2 Department of Ophthalmology, University Emergency Hospital, 169 Splaiul Independenței Street, District 5, 050098 Bucharest, Romania; 3 Department of Obstetrics and Gynecology, Faculty of Medicine, “Carol Davila” University of Medicine and Pharmacy, District 5, 020021 Bucharest, Romania; klee_ro@yahoo.com; 4 Obstetrics and Gynecology Department, Clinical Hospital of Obstetrics and Gynecology “Filantropia”, 011132 Bucharest, Romania

**Keywords:** pregnancy, corneal biomechanics, corneal hysteresis, corneal resistance factor, intraocular pressure, sex hormones, thyroid hormones

## Abstract

*Background and Objectives:* There is a clear evidence that pregnancy is associated with high production of sex hormones. During the first, second and third trimester of pregnancy, blood hormones levels increase gradually. Cells with affinity for sex hormones have been identified in different ocular tissues, such as: lid, lacrimal gland, meibomian gland, bulbar and palpebral conjunctivae, cornea, iris, ciliary body, lens, retina (retinal pigment epithelium) and choroid. This is why pregnancy is associated with changes at ocular level, involving anterior and posterior segments. Several clinical trials have been made trying to highlight changes in corneal biomechanics during pregnancy. By conducting this review, we want to evaluate both the changes in parameters that define corneal biomechanics and intraocular pressure values in pregnant. *Materials and Methods:* Following a systematic search in the literature related mainly to changes in corneal biomechanics during pregnancy, focusing on the paper published in the last decade, we included in a meta-analysis the cumulative results of three prospective comparative studies. *Results:* Important changes in corneal biomechanics (corneal hysteresis and corneal resistance factor) parameters were observed in women in the third trimester of pregnancy, but these variations were not statistically significant. Also, a decrease in intraocular pressure was mentioned in these women, but only the corneal compensation intraocular pressure showed a decrease with statistical significance. *Conclusions:* A decrease in corneal compensatory intraocular pressure was observed in pregnant women in the third trimester of pregnancy, but without other statistically significant changes resulting from the analysis of the other three parameters (corneal hysteresis, corneal resistance factor and Goldmann-correlated intraocular pressure).

## 1. Introduction

During pregnancy, women are exposed to various series of anatomical–physiological body changes. In the first weeks of pregnancy, these modifications are secondary to the fetus, placenta and uterus metabolic demands and to the increasing levels of sex hormones. Starting with the second trimester of pregnancy, the anatomical changes are caused by the mechanical action of the uterus, while the variation in sex hormone concentrations becomes much more significant [1]. These actions are completely physiological and most commonly involve multiple organs, including the eyes [2,3].

There is a clear evidence that pregnancy is associated with the high production of sex hormones. There are three large groups of sex hormones: estrogens, progestogens and androgens [4]. During the first, second and third trimester of pregnancy, blood hormone levels increase gradually, and then, postpartum, their values decrease rapidly in a few days [5,6,7].

In recent years, cells with affinity for sex hormones have been identified in different ocular tissues, such as lid, lacrimal gland, meibomian gland, bulbar and palpebral conjunctivae, cornea, iris, ciliary body, lens, retina (retinal pigment epithelium) and choroid. According to researchers, this is why pregnancy is associated with changes at the ocular level, often self-limited during it, which involves both anterior and posterior segments [8,9,10,11,12,13,14,15,16].

It is known that the main functions of the cornea are the protection against harmful factors, of any kind, from the external environment, and refraction, with the cornea being the strongest surface that can achieve refraction of light entering the eye: it represents almost 80% of the total refractive power. The stroma of the cornea constitutes approximately 90% of the thickness and is the one that provides greater rigidity to the cornea, so, from a biomechanical point of view, it may be assumed that the stroma is the layer that determines its elasticity and viscoelastic behavior [17,18]. It consists mainly of collagen fibers and extracellular matrix (proteoglycans and glycosaminoglycans) [19,20]. The CH (corneal hysteresis) reflects the corneal ability to absorb and then dissipate the energy when stress–relaxation forces are applied (applanation forces), and it indicates the viscoelastic proprieties of the cornea. On the other hand, CRF (corneal resistance factor) is an indicator of the corneal global resistance [21,22,23].

To date, specialized studies have mentioned the following changes that may occur during pregnancy due to hormonal changes that affect the structures of the cornea: increased corneal thickness, increased corneal curvature, modifications in corneal sensitivity, decreased intraocular pressure (IOP), changes in refraction, visual field defects, contact lens intolerance and variations in corneal biomechanics [24,25,26,27,28,29,30,31,32,33,34].

Some papers attest to the existence of thyroid hormone receptors in human corneal tissue [11]. The existence of these hormones in the mother’s blood during pregnancy is very important, and the development of the fetus is dependent on it [35,36,37,38,39,40,41]. Studies have shown the link between these hormones in corneal biomechanics, in the variation in intraocular pressure and in changes in corneal curvature during pregnancy [42,43,44,45].

By conducting this review based on existing cohort studies to date, we shall evaluate both changes in the parameters that define the biomechanics of the cornea and the values of intraocular pressure in women in the third trimester of pregnancy.

## 2. Materials and Methods

### 2.1. Objectives

Starting from the hypothesis that there are corneal receptors for sex hormones and thyroid hormones and based on the clear evidence that during pregnancy there is a variation in these hormones in the blood, changes in the parameters of corneal biomechanics and intraocular pressure during pregnancy have been highlighted [11,12,13]. With this review, we want to evaluate both the changes in the parameters that define corneal biomechanics (CH and CRF) and the values of intraocular pressure (IOPcc and IOPg). This will be done by comparing the aforementioned parameters for a group of third-trimester pregnant women to a group of non-pregnant women.

### 2.2. Search Strategy

Through a systematic search in specialized publications, we identified articles published between 2013 and 2020. In-depth searches have been performed in various electronic databases such as PubMed, Embase, Web of Science and Scopus, in order to identify specialized articles that comparatively analyze pregnant women.

The following keyword combinations have been introduced to identify medical papers: pregnancy & corneal biomechanics, pregnancy and corneal hysteresis, pregnancy & corneal resistance factor, pregnancy & intraocular pressure, pregnancy & sex hormones and pregnancy & thyroid hormones. In the search process, we entered two independent researchers.

The reference lists of the articles integrated in the review have been analyzed with the help of the electronic database in order to identify the eligible articles.

### 2.3. Inclusion and Exclusion Criteria

Following the detailed analysis of medical papers found on electronic platforms, we have included the studies in which healthy pregnant women in trimester one, two or three were measured using dynamic bidirectional applanation device, thus recording the changes in corneal biomechanics and intraocular pressure.

The exclusion criteria from these studies were as follows: cornea inflammation and infections history, pre-existing corneal lesions, wearing contact lenses, administration of topical eye treatment, history of eye trauma, history of eye surgery and other known eye pathology. At the same time, pregnant women with known systemic diseases and those with pregnancy-related complications were excluded from the study. Pregnant women who took oral contraceptives before the current pregnancy were also not included in the studies.

### 2.4. Data Collection, Data Analysis and Outcomes

This review is based on five prospective case–control clinical trials that included a total of 356 pregnant women and a total of 216 non-pregnant women. Pregnant women enrolled in the studies were in different weeks of gestation, thus noting their belonging to each of the corresponding trimesters of pregnancy [46,47,48,49,50].

Each person included in the study underwent a complete ophthalmic clinical examination consisting of: visual acuity measurement using the Snellen test, eye refraction, slit-lamp biomicroscopy and fundus examination. In order to quantify the intraocular pressure and the parameters related to corneal biomechanics, in all the mentioned studies, the women were measured by a non-invasive, repetitive and rapid maneuver using the Ocular Response Analyzer device (ORA, Reichert Ophthalmic Instruments, New York, NY, USA). The parameters resulting from this last investigation that were analyzed and then documented as means were the following: CH, CRF, IOPcc and IOPg.

The preparation of the data for the statistical analysis of the four compared parameters was performed with the help of the Excel 2019 program (Microsoft Office, Bucharest, Romania). The data were introduced into the statistical meta-analysis program used by The R Project software, version 4.0.4, to be analyzed and compared. We performed fixed-effect meta-analysis using the analysis packages according to the protocol. Then, the recorded values were compared using the programming language according to the raw effect size data function [50]. We used individual confidence intervals to identify statistically significant differences among the group means and determine whether the differences are practically significant.

## 3. Results

The literature search resulted in 108 articles related to corneal biomechanics during pregnancy. Thirty-six of them were selected for abstract review. After abstract reviewing, we included five trials. One trial had no control group, while another one had no specific data related to pregnancy age, so these two trials were excluded from the data analysis. Therefore, there are three trials quantitatively evaluated in our review, according to Table 1 [46,47,48,49,50].

All included studies are prospective comparative studies, and all of them analyze all parameters of cornea biomechanics. We used confidence intervals (95% CI) to assess the differences among group means. If the range of CI does not include zero, it indicates that the difference among these means is statistically significant. If the CI interval includes zero, then the difference is not statistically significant.

We included studies performed between 2013 and 2019. From the total number of pregnant women enrolled in the three studies, we selected only those who were in the third trimester of pregnancy. Thus, in our review, we analyzed a total number of 217 pregnant women and a total number of 182 non-pregnant women.

**Figure 1:**
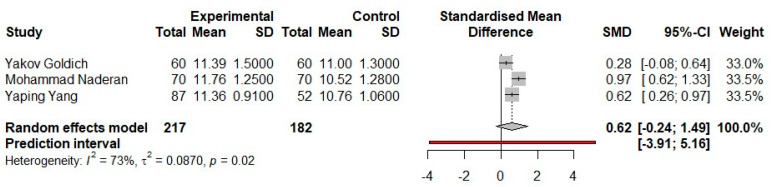
Fixed-effects meta-analysis and forest plot of the three prospective comparative trials studies on the corneal biomechanics parameter-CH-in pregnant women who are in the third trimester versus a control group of non-pregnant women. SD, standard deviation; SMD, standardized mean difference; CI, confidence interval.

The first parameter we analyzed was CH, and the results are shown in Figure 1. The studies represent an almost equal percentage value of weight, resulting in a similar influence of the three studies in the overall effect. Furthermore, the confidentiality intervals for each study are comparatively equal in length. Two of the studies show a range of 95% CI outside the value of zero, respectively, with the result that these studies individually show a statistically significant result. However, it is noticed in Yakov Goldich’s study that 95%-CI crosses the line of no effect, thus resulting in no statistical significance at the study level.

A comparative analysis for the effect size of the variable CH for each study shows how they poorly overlap, which also results from the substantial heterogeneity of the studies and from the value of *p*, which is <0.05.

Examining the meta-analytical summary, the pooled effect size value is 0.62, but the 95% CI interval passes through the value zero. Thus, the results for the analysis of the CH parameter are not statistically significant (*p* > 0.05).

**Figure 2:**
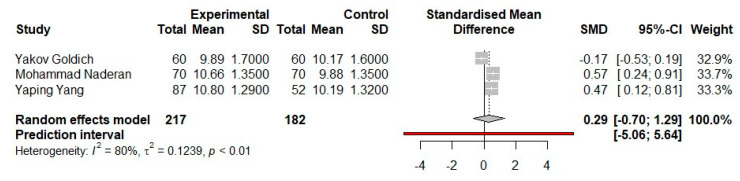
Fixed-effects meta-analysis and forest plot of the three prospective comparative trials studies on the corneal biomechanics parameter-CRF-in pregnant women who are in the third trimester versus a control group of non-pregnant women. SD, standard deviation; SMD, standardized mean difference; CI, confidence interval.

Analyzing the corneal biomechanical variable CRF (Figure 2), the influence that each individual study has on the collective result is reported as a percentage, and they are almost equal. It is noticed in the case of two of the three articles that the value of 95% CI does not reach the line of no effect. In the case of the study presented by Yakov Goldich et al., the forest plot diagram demonstrates how the 95% CI value is not statistically representative, because the confidentiality interval passes through the zero value.

The value of I^2^ indicates the level of heterogeneity represented by these three studies, and in this case, its value is 80%. The heterogeneity is thus substantial, and the value of *p* is <0.01.

Analysis of the general effect resulting from the comparison of the CRF parameter means between studies emphasizes the value of pooled effect size of 0.29, as well as the fact that the 95% CI interval reaches the line of no effect, marking the point where there is no clear difference between the groups. The 95% CI crosses the line of no effect, with the result that the analysis of the CRF value does not show statistical significance (*p* > 0.05).

**Figure 3:**
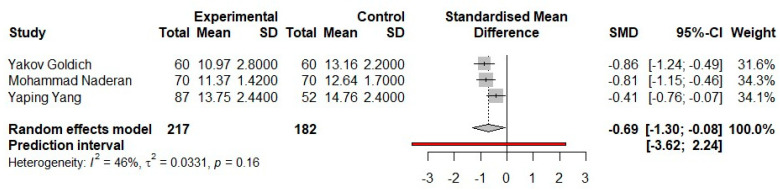
Fixed-effects meta-analysis and forest plot of the three prospective comparative trials studies on the corneal biomechanics parameter-IOPcc = in pregnant women who are in the third trimester versus a control group of non-pregnant women. SD, standard deviation; SMD, standardized mean difference; CI, confidence interval.

Figure 3 provides summary data for each study comparing the mean values of the IOPcc variable between the groups of pregnant women in the third trimester and the control group. The three studies present an almost equal-weight percentage in the case of this meta-analysis. Regarding the heterogeneity statistics of the studies, it resulted in a procedural value of I^2^ is 46% and a high *p*-value. We can conclude that the heterogeneity is not significant and that the statistical results can be further analyzed.

Analyzing the estimated effect and the 95% CI interval for each study, we notice that none of the studies overlaps with zero. The sizes of the three intervals are similar. Therefore, in conclusion, there is a statistical significance at the level of each study.

The overall effect estimate does not pass through the line of no effect, and the value of the 95% CI interval is entirely negative. Therefore, we can see that there is statistical significance at the level of meta-analysis (*p* < 0.05). The intervention is better because both the standardized mean difference and the 95% CI range are to the left of the line of no effect for the IOPcc variable.

**Figure 4:**
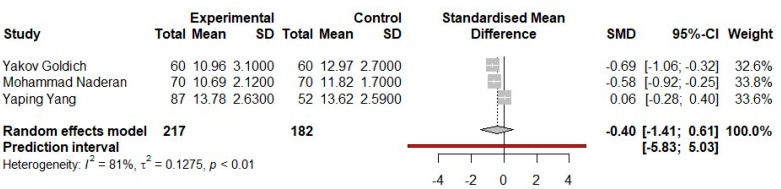
Fixed-effects meta-analysis and forest plot of the three prospective comparative trials studies on the corneal biomechanics parameter-IOPg-in pregnant women who are in the third trimester versus a control group of non-pregnant women. SD, standard deviation; SMD, standardized mean difference; CI, confidence interval.

In the case of the IOPg parameter, according to Figure 4, the 95% confidence intervals exceed the value of 0 only in the case of a single clinical study. Therefore, there is no statistical significance at study level for Yaping Yang’s paper. The other two studies show a negative 95% CI range value, so each individually has a statistical significance.

It can be seen that the weight of the studies is represented in equal percentages. Analyzing the heterogeneity of the values introduced from each study shows that there is an increased percentage of I^2^ (important heterogeneity) with a value of *p* < 0.01.

The statistical analysis of IOPg shows that there is no statistical significance at the level of the meta-analysis (*p* > 0.05), because the 95% CI of the overall effect estimate overlaps the line of no effect.

## 4. Discussion

Physiological changes in the ophthalmic system are very common during pregnancy. On the other hand, pregnancy can show pathological effects on the eye or can cause decompensation of the pre-existing medical condition. However, all these changes are often limited throughout the pregnancy, and after birth, they gradually disappear [14,15,16].

In recent years, advanced studies have concluded that at the corneal level, receptors for estrogen, progesterone, androgen and thyroid hormones were found in the nuclei of epithelial, stromal and endothelial cells [11,12,13]. Therefore, increasing the level of sex and thyroid hormones during pregnancy can lead to significant changes in the cornea.

The variation in sex hormones during pregnancy is very important. Increased blood estrogen level is detected for the first time in the first trimester (in approximately week 9 of gestation) [5] and the level of progesterone in the second trimester of pregnancy (in approximately week 20 of gestation). Shortly after delivery, estrogen and progesterone production decreases, reaching pre-conception values, comparable to those of a non-pregnant woman [6,7].

Changes in corneal biomechanics are strongly influenced by the marked increase in sex hormones, especially in the third trimester of pregnancy. The most important estrogens produced in the ovary are estradiol and estrone. Experimental studies have shown that pregnancy is associated with an increased level of estrogen that stimulates the production of prostaglandin, matrix proteinase and collagenolytic enzymes activator, therefore modulating the biomechanical properties of the cornea. High estrogen levels promote hyaluronic acid (hydrophile) in the corneal cells that can lead to an increase in corneal thickness, due to excessive hydration at this level [27,28]. In contrast, progesterone is an inhibitor of the synthesis of prostaglandin, and it can inhibit matrix degranulation, thus balancing the effect of estrogens [29,30,31].

Studies have shown that there is also a decrease in intraocular pressure during pregnancy, which is common and temporary. The mechanisms that have been stipulated in specialized studies are: increase of the outflow rate of aqueous humor secondary to high levels of sex hormones in the blood or increased corneal thickness due to corneal hydration (possibly an effect of estrogen) [32,33,34].

Thyroid function changes throughout the gestation period. During early pregnancy, the fetus is completely dependent on the mother for thyroid hormone production. At the end of the first trimester, the baby’s thyroid starts making hormones by itself [37]. The baby, however, is still dependent on the mother’s ingestion of enough iodine, which is essential for the production of thyroid hormones. In the first trimester, maternal TSH is normal or slightly decreased and then remains normal for the rest of the pregnancy. At the beginning of pregnancy, normal levels of free T4 and low or normal levels of T3 in maternal blood have been recorded [38,39,40,41].

The negative effect of estrogen on corneal biomechanics is balanced by the opposite effect of progesterone and is modulated by thyroid hormones. Following these hypotheses, specialized analysis has stated the existence of a link between the variation ib thyroid hormone level during pregnancy and the variation ib intraocular pressure, the increase in corneal thickness and the adjustment of corneal curvature [42,43,44,45].

In this review, we included three prospective clinical trials that looked at a total of 217 third-trimester pregnant women and a total of 182 non-pregnant women. With the help of the Ocular Response Analyzer (ORA) device, data related to the anterior ocular segment have been obtained from the women enrolled in each study. The following parameters of corneal biomechanics were noted for each paper: corneal hysteresis (CH), corneal resistance factor (CRF), corneal compensation intraocular pressure (IOPcc) and Goldmann-correlated intraocular pressure (IOPg) [46,47,48].

In the study presented by Yakov Goldich et al., which analyzed a group of 60 third-trimester pregnant women with a group of 60 non-pregnant women, no statistically significant difference in CH and CRF parameters was noted. In the case of intraocular pressure variation, this paper highlights a statistically significant decrease in IOPcc and IOPg in pregnant women [46].

Statistical analysis performed in the study of Mohammad Naderan et al. focused on 140 women, half of whom were third-trimester pregnant. In this paper, no statistically significant change was noted in any of the analyzed parameters (CH, CRF, IOPcc, IOPg) comparing pregnant to non-pregnant women [45].

The paper presented by Yaping Yang et al. compared a group of 52 pregnant women with a group of 170 women in all trimesters of pregnancy. Of the latter, 87 pregnant women were in the third trimester. Elevated values of the CH and CRF parameters were statistically significant after comparing pregnant women in the third trimester with non-pregnant women. A decrease in intraocular pressure values (IOPcc and IOPg, respectively) has been noted in this study following the analysis performed in this paper, but this has no statistical significance [47].

This review cumulated data from the three studies mentioned above, thus trying to show that there is indeed a variation in the four variables followed during pregnancy. Therefore, we sampled the data of the parameters CH, CRF, IOPcc and IOPg, respectively, correlated with pregnant women in the third trimester and non-pregnant women, presented in each clinical study in the form of mean values and standard deviation (SD) values. We introduced the resulting values in a meta-analysis with fixed-effect, performed through lines of code implemented using the R program of statistical meta-analysis.

From the forest plot diagrams made for the analysis of each parameter of corneal biomechanics, CH and CRF, respectively, in each case, the paper of Yakov Goldich et al. showed no statistical significance because the 95% confidence interval crosses the no-effect line. In the case of the studies presented by Mohammad Naderan et al. and Yaping Yang et al., these confidence intervals are completely to the right of the no-effect line for each of the variables studied individually; thus, these studies show statistical significance in the meta-analysis. However, the 95% CI of the overall effect of each statistical analysis is [−0.25; 1.49] for the parameter CH and [−0.70; 1.29] for the CRF parameter; thus, there is no statistical significance following the analysis of these parameters among the three studies (*p* > 0.05).

Moreover, in the case of the meta-analysis performed through the three clinical studies on the IOPg parameter, the studies of Yakov Goldich et al. and Mohammad Naderan et al. are representative in the statistics, with the values of the 95% CI intervals being [−1.06; −0.32] and [−0.92, respectively; −0.25]. However, in the case of the study presented by Yaping Yang, it does not show individual statistical significance, because the 95% CI crosses the no-effect line. The range of the values of the 95% CI ([−1.41; 0.61]) of the overall effect is statistically insignificant (*p* > 0.05).

In the current review, we have noticed further to the meta-analysis performed on the IOPcc parameter that each of the three included studies had its own statistically significant value. The confidence intervals for each nominal study showed negative values and did not reach the no-effect line, which in part was as follows: for the study of Yakov Goldich et al., the 95% CI was [−1.24; −0.49]; for the study of Mahommad Naderan et al., the 95% CI was [−1.15; −0.46]; and for the study of Yaping Yang et al., the 95% CI was [−0.76; −0.07]. Regarding the analysis of the heterogeneity of the included studies, the I^2^ test presents a percentage of 46%, so a moderate level heterogeneity, together with a value of *p* = 0.16.

Following the summary of the meta-analysis through the forest plot diagram, it can be seen how the entire 95% CI interval and the standardized mean difference (SMD) value present negative values. The 95% CI has the value of [−1.30; −0.08], thus showing that it does not overlap the no-effect line, being to its left in totality, thus leading to a statistically significant result (*p* < 0.05). The SMD value is also negative at −0.69. Following the meta-analysis performed using the three clinical trials, we can conclude that the value of corneal compensation intraocular pressure is lower by 0.69 mmHG in pregnant women in the third trimester compared to non-pregnant women.

### Limitations

Several limitations of this review have to be emphasized. A limited number of prospective case–control studies comparing corneal biomechanics and intraocular pressure values with ORA between third-trimester pregnant women and non-pregnant women have been identified. Moreover, a limited total number of cases were enrolled in the three papers, making the statistical analysis present a possibly low power in identifying the real variations in the studied parameters.

## 5. Conclusions

In the meta-analysis performed by entering all the statistical data from three clinical trials, a statistically significant value was noted for the IOPcc parameter, resulting in a decrease in corneal compensation intraocular pressure in the third trimester of pregnancy between 0.08 mmHg and 1.30 mmHg, averaging 0.69 mmHg. With this result, we must ask ourselves if it is also clinically significant, which may be the cause and the consequences of this decrease in IOPcc.

Although increases in CH and CRF values along with decreases in IOPg have been noticed for each paper, they are not statistically significant in all cases. From the data presented in the forest plot, no statistically significant values are identified following the analysis of the three parameters introduced, namely CH, CRF and IOPg.

## Figures and Tables

**Table 1:** Characteristics of Each Trial Included in the Study.

Source	No. Pregnant Women in the Third Trimester/No. Control	Type of Study	Objective	Conclusions
Goldich Y. et al. [46] (2014)	60/60	prospective case–control	Quantification of changes in the anterior anatomical segment, in the properties of corneal biomechanics and intraocular pressure values during pregnancy.	Decreases in IOP and a steeper cornea were observed during pregnancy. There were no statistically significant changes related to CH and CRF values, volume and depth of the anterior chamber, variations in iridocorneal angle and corneal thickness.
Naderan M. et al. [45] (2018)	70/70	prospective case–control	Evaluation of changes in corneal topography and corneal biomechanics during pregnancy.	Analyzing the parameters of corneal topography and corneal biomechanics, ocular changes during pregnancy return to baseline in the postpartum period.
Yang Y. et al. [47] (2020)	87/52	prospective case–control	Assessment of changes in corneal biomechanics and IOP values in pregnant Chinese women.	Statistically significant changes in corneal biomechanics parameters were observed with decreased corneal intraocular pressure pressure and increases in CH and CRF parameters. No significant variations of the IOPg parameter were observed.

## Data Availability

There are no original data to make available for this review paper.

## Abbreviations

ORA	Ocular Response Analyzer
CH	Corneal Hysteresis
CRF	Corneal Resistance Factor
IOPcc	Corneal Compensation Intraocular Pressure
IOPg	Goldmann-Correlated Intraocular Pressure
